# Retraction statement: Long non‐coding RNA PGM5‐AS1 promotes epithelial‐mesenchymal transition, invasion and metastasis of osteosarcoma cells by impairing miR‐140‐5p‐mediated FBN1 inhibition

**DOI:** 10.1002/1878-0261.13482

**Published:** 2023-07-20

## Abstract

The above article, published online on 19 Aug 2020 in Wiley Online Library (wileyonlinelibrary.com), has been retracted by agreement between the journal's Editor‐in‐Chief, Kevin Ryan, FEBS Press, and John Wiley and Sons Ltd. The retraction has been agreed following an investigation into concerns raised by a third party, which revealed inappropriate duplications of image panels within the article (2 panels of fig. 4G). Compelling raw data was not available for either the microscopy images, nor the Western blots across figs 2, 3, 5–8. Thus, the editors consider the conclusions of this manuscript substantially compromised.

Reference

1 Liu W, Liu P, Gao H, Wang X, Yan M. Long non‐coding RNA PGM5‐AS1 promotes epithelial‐mesenchymal transition, invasion and metastasis of osteosarcoma cells by impairing miR‐140‐5p‐mediated FBN1 inhibition. *Mol Oncol*. 2020;**14**:2660–2677. https://doi.org/10.1002/1878-0261.12711

The above article, published online on 19 Aug 2020 in Wiley Online Library (wileyonlinelibrary.com), has been retracted by agreement between the journal's Editor‐in‐Chief, Kevin Ryan, FEBS Press, and John Wiley and Sons Ltd. The retraction has been agreed following an investigation into concerns raised by a third party, which revealed inappropriate duplications of image panels within the article (2 panels of fig. 4G). Compelling raw data was not available for either the microscopy images, nor the Western blots across figs 2, 3, 5–8. Thus, the editors consider the conclusions of this manuscript substantially compromised.

**Reference**

1 Liu W, Liu P, Gao H, Wang X, Yan M. Long non‐coding RNA PGM5‐AS1 promotes epithelial‐mesenchymal transition, invasion and metastasis of osteosarcoma cells by impairing miR‐140‐5p‐mediated FBN1 inhibition. *Mol Oncol*. 2020;**14**:2660–2677. https://doi.org/10.1002/1878-0261.12711

